# Impacts of the COVID-19 pandemic on excess deaths: descriptive study, Pernambuco, 2020-2022

**DOI:** 10.1590/S2237-96222025v34e20240286.en

**Published:** 2025-09-01

**Authors:** Hélder Limeira Campos, Gabriel Jesus Alves Fernandes, Daphne Galvão de Sousa, Paloma Luna Maranhão Conrado, Évelyn de Oliveira Campos, Ricardo Augusto Barros dos Santos, Polyana Felipe Ferreira da Costa, Carolina Maria da Silva, George Alessandro Maranhão Conrado, Pauliana Valéria Machado Galvão

**Affiliations:** 1 Universidade de Pernambuco, Faculdade de Medicina, Serra Talhada, PE, Brazil; 2 Universidade de Pernambuco, Faculdade de Ciências Médicas, Recife, PE, Brazil

**Keywords:** COVID-19, Pandemics, Mortality, Epidemiology, Descriptive, Information Systems, COVID-19, Pandemias, Mortalidad, Epidemiología Descriptiva

## Abstract

**Objective:**

To estimate the excess deaths during the COVID-19 pandemic in Pernambuco between 2020 and 2022.

**Methods:**

This descriptive study used data from the Mortality Information System (SIM). The excess deaths, expressed as the observed-to-expected deaths ratio, and the P-score, expressed as the proportional excess deaths, were evaluated. The estimated deaths used historical data from 2015 to 2019 based on linear regression models.

**Results:**

The highest proportional excess mortality for 2020 was evidenced in the Regional Health Management Department I, III, and IV (28.0%, 25.9%, and 18.1%); for 2021, in the Regional Health Management Departments I, XII and VIII (35.4%, 24.7% and 23.2%); and for 2022, in the Regional Health Management Departments XI, IV and VII (20.1%, 19.1% and 16.3%).

**Conclusion:**

The distribution of excess mortality in Pernambuco varied during the pandemic years and highlighted the inland spread of COVID-19-related deaths.

## Resumen

**Objetivo:**

Estimar el exceso de mortalidad durante la pandemia de COVID-19 en Pernambuco entre 2020 y 2022.

**Métodos:**

Este fue un estudio descriptivo utilizando datos del Sistema de Información de Mortalidad. Se evaluaron el exceso de mortalidad, expresado como la relación de muertes observadas a esperadas, y el P-score, expresado como el exceso proporcional de muertes. Para las muertes estimadas se utilizaron datos históricos de 2015 a 2019, basados en modelos de regresión lineal.

**Resultados:**

El mayor exceso proporcional de mortalidad para 2020 se evidenció en las oficinas regionales de salud I, III y IV (28,0%, 25,9% y 18,1%); para 2021, en las oficinas regionales de salud I, XII y VIII (35,4%, 24,7% y 23,2%); y para 2022, en las oficinas regionales de salud XI, IV y VII (20,1%, 19,1% y 16,3%).

**Conclusión:**

La distribución del exceso de mortalidad en Pernambuco varió durante los años de la pandemia y mostró el proceso de cambio hacia regiones del interior de mortalidad por COVID-19.

**Ethical aspects**

This research used public domain anonymized databases.

## Introduction

The COVID-19 pandemic was the biggest health disaster of the century (1,2). Globally, there have been around 770 million people infected and around 6.9 million deaths related to the disease (3). In addition to the directly associated repercussions, the pandemic has been correlated with a significant increase in respiratory, thromboembolic, cardiovascular, and renal disease incidence (4). Furthermore, it intensified pre-existing medical conditions and caused psychological disorders such as anxiety and depression, as well as causing substantial financial losses. 

These effects have triggered secondary consequences, which could result in further losses of human life (5). Accurate and precise estimates of deaths due to COVID-19 in low- and middle-income countries are not easily obtained, mainly due to the difficulties of monitoring epidemiological surveillance (6).

In Brazil, the significant effects of the pandemic were influenced by a lack of strategic planning for controlling and mitigating outbreaks and public health emergencies, a lack of integration between the three spheres of government (federal, state, and municipal), and harmful political scenarios (1). Factors such as the scarcity of resources, non-compliance with isolation measures, and the challenges of vaccination (5) have led to the country ranking sixth in the world in terms of the number of cases and second in the number of deaths (3).

The media coverage and much of the scientific debate in Brazil have only considered reports of deaths from COVID-19, despite the obvious limitations of such data for comparing states with different population sizes, as well as underestimating its impact on deaths (7). Therefore, assessing the effects of COVID-19 has become a challenge, which is why calculating the excess deaths as a tool to estimate the real impact of the pandemic on the general mortality of the population is recommended (8). 

Excess deaths are the difference in the total number of deaths observed in an epidemiological crisis compared to the deaths expected if the crisis had not occurred (9). This indicator allows us to estimate the direct and indirect effects of deaths in epidemics/pandemics, assists the decision-making process at different stages of the event (9,10), and allows for a more complete assessment of the negative consequences for the health system and society (10). 

In the national overview, it is evident that each region of the country has faced different realities concerning the pandemic, highlighting the need for different approaches to understanding, in an integrated and broad way, the impacts of COVID-19 in Brazil, considering the regional inequality that affects the country.

During the pandemic, Pernambuco was the Brazilian state with the highest increase in the poverty rate, registering an increase of 8.14%, which resulted in around 1.6 million people living on a monthly income of less than BRL 500.00 (11). The state was third in the Northeast in the number of confirmed cases (1,235,663) and deaths from COVID-19 (23,241) (12), which reflected the complexity of the socio-economic and epidemiological impacts of the pandemic in the region. However, there are still few studies that evaluate the impact of the pandemic in Pernambuco, which is an essential analysis for understanding the disease patterns and its consequences in the state and other regions of Brazil. 

This study aimed to estimate the excess deaths during the COVID-19 pandemic in Pernambuco between 2020 and 2022. For this purpose, it compared deaths in the state during 2020-2022 with the average of the last years of the pre-pandemic period (2015-2019).

## Methods

### Design

This was a descriptive study involving the deaths reported in the Mortality Information System (SIM) between 2015 and 2022, with Pernambuco as the unit of analysis. The data had been updated by December 2022, consolidated in SIM by April 2024, and made available from June 2024. The data was extracted on July 15, 2024.

### Setting

Pernambuco comprises 185 municipalities, divided into four health macro-regions: Metropolitan Region, Agreste, Sertão and São Francisco Valley and Araripe. The State Health Department coordinates these municipalities in 12 Regional Health Management Departments. These Regional Health Management Departments are located in Recife (Regional Health Management Department I), Limoeiro (Regional Health Management Department II), Palmares (Regional Health Management Department III), Caruaru (Regional Health Management Department IV), Garanhuns (Regional Health Management Department V), Arcoverde (Regional Health Management Department VI), Salgueiro (Regional Health Management Department VII), Petrolina (Regional Health Management Department VIII), Ouricuri (Regional Health Management Department IX), Afogados da Ingazeira (Regional Health Management Department X), Serra Talhada (Regional Health Management Department XI) e Goiana (Regional Health Management Department XII) (13).

### Participants

All deaths notified in the SIM between 2015 and 2022 were included, covering all causes of death, age groups, and both sexes registered in Pernambuco. 

Deaths whose records were inconsistent or lacked essential information, such as data identifying the underlying or associated causes of death, were excluded. The analysis did not consider deaths occurring outside Pernambuco or not notified in the SIM. 

### Variables

The variables analyzed included data related to general deaths in Pernambuco and deaths associated with COVID-19, namely: sex (male, female), underlying cause of death, year and epidemiological week of death, and the regional health management department of Pernambuco where the death was registered.

The underlying cause of death was analyzed using the 10th Edition of the International Statistical Classification of Diseases and Related Health Problems (ICD-10), considering all the causes described in its 22 chapters and covering all the categories and codes registered.

Deaths considered to be related to COVID-19 included both those in which COVID-19 was recorded as the underlying cause of death and those in which COVID-19 was mentioned as a contributing or associated cause on the death certificate, under ICD-10 B34.2 (coronavirus infection, unspecified site) (14).

### Data sources and measurement

The information on deaths was obtained from SIM. 

To ensure the robustness and transparency of the findings, the final SIM data for the period 2015-2022 was used, consolidated up to March 2023. The data was only collected once the final version had been made available by SIM, guaranteeing the completeness of the information and minimizing the possible impact of delays in notification. This ensured the accuracy of the results presented for the period analyzed.

Excess deaths were measured as the difference between the deaths actually reported during a given period and the deaths expected during that period if the COVID-19 pandemic had not occurred. The following formula was used: *Excess deaths=number of reported deaths-number of expected deaths* (8,15).

For comparison purposes, the P-score was used instead of presenting excess deaths since the gross number of excess deaths gives a sense of scale but is less comparable because they can have large differences in population size and expected annual deaths.

This metric is calculated as: *P-score=[(reported deaths-projected deaths)/projected deaths*]x100 (4).

The observed-to-expected deaths ratio was calculated for each Regional Health Management Department studied (8). This calculation provided a detailed analysis of variations in mortality over time and in different regions of the state and contributed to a more comprehensive understanding of mortality patterns during the period under analysis.

Historical data from 2015 to 2019 was used to estimate expected mortality.

### Bias control

The possibility of information bias was considered, and this was due to the type of data used in this study. The number of deaths reported may not include all the situations that occurred due to the limitations of how information on death is produced. Not all locations have the capacity to properly record and report all deaths due to the lack of hospitals, health staff, and infrastructure to record them. In the context of the pandemic, delays in mortality reporting have been frequent, and data can be provisional and incomplete in the weeks, months, or even years after a death has occurred.

The possibility of bias caused by incomplete data was also considered. Incomplete data can lead to wrong conclusions, reduced accuracy, and limitations in the applicability of the results.

Another limitation may be connected to important differences between regional health management departments, such as health care infrastructure, death registries, and surveillance processes. The pandemic has emphasized the operational difficulties of both care and the registration and investigation of deaths due to the overload of the systems and professionals in charge. It is important to consider that the pandemic may have masked the effect on mortality of other epidemiological events (outbreaks, epidemics, and endemic diseases).

### Study size

This was a census-based study, and sample calculation was not used.

### Statistical methods

Expected mortality was estimated using the regression model separately for each Regional Health Management Department for the entire state of Pernambuco:

D_t_,_Y_= α_t_+β∙Y

Where D_t_,_Y_ corresponded to the number of deaths observed during a week t in year Y; β corresponded to a slope over the years; ε ~ Ν(0,σ^2^) represented separate intercepts (fixed effects) for each week (month/quarter); and denoted Gaussian noise. This model was able to represent the seasonal variation in mortality and the annual trend in recent years due to changes in population structure or socio-economic factors (4).

As Brazil provides weekly data, the model was adjusted using weeks 1-52 since week 53 will only happen in a few years (including 2020). Therefore, the baseline for week 53 was considered the same as the value obtained for week 52 (4). This study used the same baseline for the years examined (2020-2022). 

The data was statistically analyzed using R software, version 4.3.3. 

## Results

The frequency of deaths in Pernambuco was shown for each Regional Health Management Department (Table 1). During the five years preceding the outbreak of the COVID-19 pandemic (2015-2019), the average number of deaths in the state was 63,778 (±1,895). 

**Table 1:** All-cause and COVID-19 deaths reported by regional health management department and year. Pernambuco, 2015-2022

Regional health Management department	2015	2016	2017	2018	2019	2020		2021		2022	
						All-cause	COVID-19	All-cause	COVID-19	All-cause	COVID-19
I - Recife	27,619	29,615	28,860	27,704	28,778	36,306	6,490	38,405	6,858	31,810	1,008
II - Limoeiro	4,242	4,354	4,375	4,219	4,329	4,882	578	5,314	838	4,746	120
III - Palmares	3,792	4,160	3,918	3,746	3,827	4,755	579	4,557	602	4,315	106
IV - Caruaru	9,991	10,601	9,743	9,399	9,618	10,899	1,314	11,202	1,563	10,991	407
V - Garanhuns	3,774	4,109	3,968	3,748	3,911	4,333	333	4,624	547	4,401	126
VI - Arcoverde	2,587	2,600	2,480	2,397	2,628	2,800	171	2,950	349	2,895	89
VII - Salgueiro	831	925	919	870	894	1,022	83	1,041	160	1,056	20
VIII - Petrolina	2,332	2,418	2,534	2,585	2,694	2,919	199	3,434	516	3,223	98
IX - Ouricuri	2,015	2,101	1,980	2,026	2,066	2,269	212	2,500	462	2,295	80
X - Afogados	1,348	1,475	1,369	1,356	1,430	1,605	144	1,708	227	1,579	43
XI - Serra Talhada	1,570	1,806	1,627	1,488	1,614	1,810	155	1,845	255	1,852	53
XII - Goiana	2,211	2,463	2,339	2,261	2,252	2,591	338	2,791	397	2,577	85
Total	62,312	66,627	64,112	61,799	64,041	76,191	10,596	80,371	12,774	71,740	2,235

**Figure 1:**
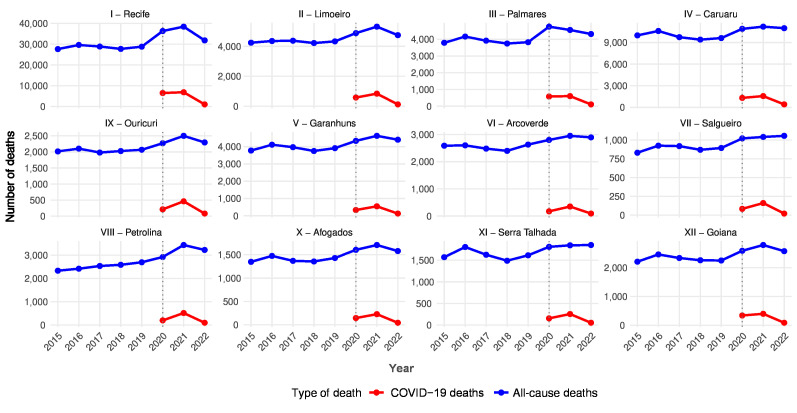
Evolution of all-cause and COVID-19 deaths by Regional Health Management Department, Pernambuco, 2015-2022

Using the regression analysis, Pernambuco’s baseline of expected deaths in 2020 was 63,212 deaths. Based on this calculation, the results showed that the state faced death counts of 20.9%, 27.5%, and 13.8% higher than expected during the years studied. The pattern highlighted by the data reflects that 2021 showed proportionally higher excess deaths than the year the pandemic began, in contrast to the decrease in 2022. When analyzing this behavior by Regional Health Management Department, the patterns found in the state were repeated, maintaining proportionality relative to the population size of each department. (Figure 1).

The observed-to-expected deaths ratio was higher for the Regional Health Management Departments I and III in 2020; I and XII in 2021; and IV, VI, VII, VIII, XI, and XII in 2022 (Table 2). These results indicated a change in the distribution pattern of excess deaths over the years and showed mortality’s shifting process toward inland regions. This information was reinforced by the P-score findings (Table 2). The highest proportional excess mortality, provided by the P-score, was observed in the Regional Health Management Departments I, III, and IV in 2020; I, XII, and VIII in 2021; and XI, IV, and VII in 2022.

**Table 2:** Observed-to-expected deaths ratio and P-score by year. Pernambuco, 2020-2022

Regional health management department	Observed-to-expected deaths ratio			P-score		
	2020	2021	2022	2020	2021	2022
I - Recife	1.3	1.4	1.1	28.0	35.4	12.2
II - Limoeiro	1.1	1.2	1.1	13.2	23.2	10.0
III - Palmares	1.3	1.2	1.1	25.9	20.7	14.3
IV - Caruaru	1.2	1.2	1.2	18.1	21.4	19.1
V - Garanhuns	1.1	1.2	1.1	11.4	18.9	13.2
VI - Arcoverde	1.1	1.2	1.2	11.0	17.0	14.8
VII - Salgueiro	1.1	1.2	1.2	12.5	14.6	16.3
VIII - Petrolina	1.1	1.2	1.2	4.8	23.3	15.7
IX - Ouricuri	1.1	1.2	1.1	11.4	22.7	12.7
X - Afogados	1.2	1.2	1.1	15.6	23.0	13.7
XI - Serra Talhada	1.2	1.2	1.2	17.3	19.6	20.1
XII - Goiana	1.2	1.3	1.2	15.7	24.7	15.1
Total	1.2	1.3	1.1	20.9	27.5	13.8

The distribution of deaths between the male and female populations, considering the causes and regional health management departments, was observed with the highest frequency always occurring in the male population (Table 3).

**Table 3:** Sex distribution of all-cause and COVID-19 deaths reported by regional health management department and year. Pernambuco, 2020-2022

Regional health management department	2020		2021		2022	
	All-cause	COVID-19	All-cause	COVID-19	All-cause	COVID-19
**Male population**						
I - Recife	19,303	3,451	19,973	3,591	16,618	513
II - Limoeiro	2,784	317	2,891	436	2,578	61
III - Palmares	2,737	330	2,529	319	2,403	51
IV - Caruaru	6,007	717	6,156	822	6,010	216
V - Garanhuns	2,412	175	2,583	300	2,367	63
VI - Arcoverde	1,568	86	1,661	191	1,601	55
VII - Salgueiro	602	38	584	93	567	11
VIII - Petrolina	1,686	119	1,994	287	1,854	56
IX - Ouricuri	1,273	130	1,414	247	1,338	49
X - Afogados	893	82	936	123	898	25
XI - Serra Talhada	997	79	1,033	136	1,036	25
XII - Goiana	1,494	189	1,556	200	1,465	40
Total	41,756	5,713	43,310	6,745	38,735	1,165
**Female population**						
I - Recife	16,991	3,039	18,425	3,267	15,185	495
II - Limoeiro	2,098	261	2,423	402	2,167	59
III - Palmares	2,016	249	2,027	283	1,912	55
IV - Caruaru	4,889	597	5,039	741	4,975	191
V - Garanhuns	1,920	158	2,039	247	2,034	63
VI - Arcoverde	1,229	85	1,288	158	1,293	34
VII - Salgueiro	420	45	457	67	489	9
VIII - Petrolina	1,232	80	1,436	229	1,368	42
IX - Ouricuri	996	82	1,084	215	956	31
X - Afogados	712	62	772	104	681	18
XI - Serra Talhada	812	76	811	119	816	28
XII - Goiana	1,097	149	1,234	197	1,112	45
Total	34,412	4,883	37,035	6,029	32,988	1,070

The observed-to-expected male deaths ratio concerning the regional health management departments was similar to that of Pernambuco, except for 2021 when the ratios were higher for Regional Health Management Departments I, V, and IX (Table 4). As indicated by the P-score, Regional Health Management Departments I, III, and VII recorded the highest excess deaths in 2020; I, IX, and V in 2021; and XI, IX, and VI in 2022. Last year, all the departments with the highest figures were in the state’s Sertão Region. The Regional Health Management Department VIII (Petrolina) had the lowest proportion of excess deaths for the male population. 

**Table 4:** Sex distribution of the observed-to-expected deaths ratio and P-score by year. Pernambuco, 2020-2022

Regional health management department	Observed-to-expected deaths ratio			P-score		
	2020	2021	2022	2020	2021	2022
**Male population**						
I - Recife	1.3	1.3	1.1	28.5	33.0	10.7
II - Limoeiro	1.1	1.2	1.1	14.4	18.8	6.0
III - Palmares	1.3	1.2	1.1	25.3	15.7	10.0
IV - Caruaru	1.2	1.2	1.2	17.0	19.9	17.1
V - Garanhuns	1.2	1.3	1.1	16.3	24.5	14.1
VI - Arcoverde	1.2	1.2	1.2	14.9	21.7	17.3
VII - Salgueiro	1.2	1.2	1.1	20.7	17.1	13.7
VIII - Petrolina	1.0	1.2	1.1	3.9	22.9	14.3
IX - Ouricuri	1.1	1.3	1.2	12.3	24.7	18.0
X - Afogados	1.1	1.2	1.1	11.8	17.2	12.4
XI - Serra Talhada	1.2	1.2	1.2	16.8	21.1	21.4
XII - Goiana	1.2	1.2	1.2	17.1	22.0	14.9
Total	1.2	1.2	1.1	20.7	25.2	12.0
**Female population**						
I - Recife	1.3	1.4	1.1	27.5	38.2	13.9
II - Limoeiro	1.1	1.3	1.2	11.8	29.1	15.4
III - Palmares	1.3	1.3	1.2	26.8	27.5	20.3
IV - Caruaru	1.2	1.2	1.2	19.6	23.3	21.7
V - Garanhuns	1.1	1.1	1.1	5.8	12.4	12.1
VI - Arcoverde	1.1	1.1	1.1	6.4	11.5	11.9
VII - Salgueiro	1.0	1.1	1.2	2.6	11.6	19.4
VIII - Petrolina	1.1	1.2	1.2	5.9	23.5	17.6
IX - Ouricuri	1.1	1.2	1.1	10.4	20.1	5.9
X - Afogados	1.2	1.3	1.2	20.7	30.9	15.4
XI - Serra Talhada	1.2	1.2	1.2	17.9	17.8	18.5
XII - Goiana	1.1	1.3	1.2	13.9	28.1	15.5
Total	1.2	1.2	1.1	20.3	29.5	15.4

For females, the death ratio behaved very similarly to that observed for the general population, except for 2022. In relation to the P-score, the highest proportions of excess deaths were identified in the Regional Health Management Departments I, III, and X in 2020; I, X, and II in 2021; and IV, III, and VII in 2022 (Table 4).

## Discussion

The COVID-19 pandemic has had a major impact on public health, and notably, the period corresponding to the peak incidence of cases has led to an increase in the number of deaths globally (2). In this context, Brazil is ranked second in terms of the number of deaths caused by the disease, ahead of the United States (3).

This study carried out in Pernambuco found excess deaths during the period analyzed. This fact is attributed, above all, to the COVID-19 pandemic, which has emerged as a new factor in mortality. These data corroborate ecological studies in other regions of the country (8,14).

This study found that the first two years of the pandemic showed a significant increase in deaths in the Regional Health Management Departments of Pernambuco, generally in an upward pattern. There was an exception concerning the Regional Health Management Department III (Palmares), which, throughout the pandemic period, showed a decrease in the number of deaths after 2020. This result aligns with the pandemic’s most critical phases: high transmission rates, the emergence of new variants, and the overloading of health systems (16).

Excess mortality is a more comprehensive measure of the total impact of the pandemic on mortality compared to the number of confirmed deaths from COVID-19. This occurs because it captures not only confirmed deaths but also those that were not accurately diagnosed and reported, as well as deaths from others that are associated with interruptions and general crisis conditions (8,14,15). 

After the critical pandemic period, there was a significant drop in excess deaths in 2022, mainly in Regional Health Management Departments VII and XI (Salgueiro and Serra Talhada). This decrease was possibly the result of a phased vaccination, prioritizing the immunization of people belonging to groups with a higher risk of morbidity and mortality (17). Further efforts are needed to understand why the listed Regional Health Management Departments have sustained this growth pattern. 

Observation revealed that the observed-to-expected deaths ratio was higher for the Regional Health Management Departments I and III in 2020, I and XII in 2021, and IV, VI, VII, VIII, XI, and XII in 2022 (Table 2). These results indicated a change in the distribution pattern of excess deaths over the years. They emphasized the death’s shifting process toward inland regions, demonstrating the progressive displacement of the pandemic’s impact from metropolitan regions to inland areas, related to increased population mobility, poorer health infrastructure, and difficulties in implementing control and surveillance measures (8).

When analyzing the timeline of excess deaths caused by COVID-19 in Pernambuco, a greater occurrence of deaths was observed in the Metropolitan Region in 2020, confirming studies showing a higher infection incidence in more densely populated regions (18). However, in 2021, there was an intensification of deaths in the state’s inland regions, mainly in the area corresponding to the Regional Health Management Departments VIII and IX. This inward spread reflects a characteristic of the inland region of Pernambuco, with areas that are home to populations in situations of vulnerability and extreme poverty, but which present major obstacles to being reached by public policies (18). 

Specifically, in the case of the Regional Health Management Departments VIII and IX, a possible conditioning factor for the increase in excess deaths was the population growth that has occurred in recent years, given that in the 2010 Population Census, the population covered by the Regional Health Management Department VIII was 434,713.0 inhabitants. In 2022, the Population Census counted 531,221.0, representing an increase of 96,508.0 (22.2%). There was also a population increase in the Regional Health Management Department IX, from 328,173 to 338,050 inhabitants, representing an increase of 9,877 (3.0%). 

Comparing the number of COVID-19 deaths between different regions has been challenging because these can be affected by each location’s testing capacity and reporting policy (19). However, global ecological studies have shown how environmental, demographic, and geographical factors, as well as the political-legal dimension, contribute to the occurrence, morbidity, and mortality of COVID-19 (20). 

Although the territorial areas of the Sertão, São Francisco Valley and Araripe are larger than those of the Metropolitan Region, it is necessary to consider that population density and a slowdown in the urbanization process have interfered with the epidemiological context of the inland region of the state, despite having fewer and less qualified healthcare resources. However, interpreting these results requires caution, as projections based on linear trends can overestimate or underestimate the real values, especially in regions with rapid population growth (21). 

The findings of this study identified that excess deaths were higher among men than among women, as previously observed (1,22).

Mortality from COVID-19 tends to be higher in males, which confirms the results of this study and can be explained by a combination of immunological, genetic, hormonal, and social factors (22). From an immunological point of view, men have lower levels of immunoglobulin G in the early stages of the disease, which can contribute to worse outcomes (23). Genetically, the X chromosome expresses genes that are essential for immunity, including those involved in cytokine signaling and Toll-like receptors, as well as the ACE2 gene, a receptor for SARS-CoV-2, whose expression can be influenced by the escape of X inactivation, resulting in sex differences in ACE2 expression and increasing vulnerability to the virus (24,25). Concerning male sex hormones, although the effects are still uncertain, low testosterone levels have been associated with worse clinical outcomes due to the role of this hormone in regulating inflammation and influencing the expression of TMPRSS2, a protease essential for viral entry (26,27). Socially, gender influences can act as social and psychological modifiers of the disease presentation, with emphasis on the lower demand for health services and low adherence to self-care among men (28).

This study faced limitations inherent to the use of secondary data, such as underreporting of deaths, possible biases related to variability in diagnostic and reporting capacity, and differences in infrastructure between regions. This can distort comparisons of excess deaths between Regional Health Management Departments, similar to what has been observed in other national studies (28,29).

Other possible limitations stem from the lack of control of seasonal factors or overlapping epidemics that may have contributed to increased deaths in certain periods. The lack of stratification by age group and risk group may also hide important differences in mortality patterns, and future studies are needed to fill these gaps.

Continuous monitoring of cases and health surveillance, especially in the epidemiological field, are essential for protecting the population. It allows for the early detection of outbreaks, the identification of risk factors, the analysis of trends, and the implementation of rapid and effective interventions that prevent the spread of diseases and reduce fatalities (5), which are fundamental aspects of the COVID-19 context. 

Given the data presented, evidence suggests that COVID-19 has had a significant impact on the health of the population of Pernambuco, with a significant excess deaths during the most critical periods of the pandemic, when, due to the collapse of health systems, there were deaths directly and indirectly associated with COVID-19. Excess deaths are an essential indicator for monitoring the impact of new health emergencies, intending to define priorities and appropriate public health measures, especially in regions with deficiencies in efficient diagnosis and notification systems.

Future studies could explore more in-depth analysis by incorporating geospatial data, population mobility patterns, and the pandemic’s long-term effects on mortality and chronic health conditions.

## Data Availability

The databases used in the research are available at: 0.5281/zenodo.13738412.
