# Supplementary material for: Impacts of the COVID-19 pandemic on excess deaths: descriptive study, Pernambuco, 2020-2022
Source: Epidemiol Serv Saude. 2025 Sep 1;34:e20240286. doi: 10.1590/S2237-96222025v34e20240286.en (PMC12404601; doi:10.1590/S2237-96222025v34e20240286.en)
Supplement: Translation

# Translation: Impactos da pandemia de covid-19 no excesso de óbitos: estudo descritivo, Pernambuco, 2020-2022

**DOI:** 10.1590/S2237-96222025v34e20240286.pt

**Authors:** Hélder Limeira Campos, Gabriel Jesus Alves Fernandes, Daphne Galvão de Sousa, Paloma Luna Maranhão Conrado, Évelyn de Oliveira Campos, Ricardo Augusto Barros dos Santos, Polyana Felipe Ferreira da Costa, Carolina Maria da Silva, George Alessandro Maranhão Conrado, Pauliana Valéria Machado Galvão

**Affiliations:** 1 Universidade de Pernambuco, Faculdade de Medicina, Serra Talhada, PE, Brasil; 2 Universidade de Pernambuco, Faculdade de Ciências Médicas, Recife, PE, Brasil

**Keywords:** Covid-19, Pandemias, Mortalidade, Epidemiologia Descritiva, Sistemas de Informação

## Abstract

**Objetivo:**

Estimar o excesso de óbitos durante a pandemia de covid-19 em Pernambuco entre 2020 e 2022.

**Métodos:**

Tratou-se de estudo descritivo utilizando dados do Sistema de Informações sobre Mortalidade. Foram avaliados o excesso de óbito, expresso pela razão entre óbitos observados e esperados, e o P-score, expresso pelos excessos de óbitos proporcionais. As mortes estimadas empregaram os dados históricos de 2015 a 2019, a partir de modelos de regressão linear.

**Resultados:**

Os maiores excessos de mortalidade proporcional para 2020 foram evidenciados nas I, III e IV regionais de saúde (28,0%, 25,9% e 18,1%); para 2021, nas I, XII e VIII regionais de saúde (35,4%, 24,7% e 23,2%); e para 2022, nas XI, IV e VII regionais de saúde (20,1%, 19,1% e 16,3%).

**Conclusão:**

A distribuição do excesso de mortalidade em Pernambuco variou durante os anos de pandemia e evidenciou o processo de interiorização da mortalidade por covid-19.

**Aspectos éticos**

Esta pesquisa utilizou bancos de dados de domínio público e anonimizados.

## Introdução

A pandemia da covid-19 foi o maior desastre sanitário do século (1,2). Em escala global, foram registradas cerca de 770 milhões de pessoas contaminadas e em torno de 6,9 milhões de óbitos relacionados à doença (3). Além das repercussões diretamente associadas, a pandemia foi correlacionada ao aumento significativo na incidência de afecções respiratórias, tromboembólicas, cardiovasculares e renais (4). Ademais, exacerbou condições médicas preexistentes e ocasionou transtornos psicológicos, como ansiedade e depressão, além de ter provocado prejuízos financeiros substanciais. 

Esses efeitos têm desencadeado consequências secundárias, que podem resultar em mais perdas de vidas humanas (5). Estimativas precisas e acuradas das mortes devido à covid-19 em países de baixa e média renda não são facilmente obtidas, principalmente devido às dificuldades de monitoramento da vigilância epidemiológica (6).

No Brasil, os efeitos da pandemia foram acentuados por falta de planejamento estratégico para controle e mitigação de surtos e emergências de saúde pública, falta de integração das três esferas de governo (federal, estadual e municipal) e devido aos cenários políticos nocivos (1). Fatores como a escassez de recursos, o descumprimento das medidas de isolamento e os desafios em relação à vacinação (5) favoreceram com que o país ocupasse a sexta posição mundial em número de casos e a segunda em número de óbitos (3).

A cobertura da mídia e grande parte do debate científico no Brasil consideraram apenas as notificações de óbitos por covid-19, apesar das limitações óbvias de tais dados para comparar estados com diferentes tamanhos populacionais, além de subestimar sua repercussão nas mortes (7). A avaliação dos efeitos do covid-19 tornou-se, por conseguinte, um desafio, motivo pelo qual recomenda-se o cálculo do excesso de óbitos como ferramenta para estimar o real impacto da pandemia na mortalidade geral da população (8). 

O excesso de óbitos é definido como a diferença no número total de mortes observadas em uma crise epidemiológica comparado com as mortes esperadas caso a crise não tivesse acontecido (9). Esse indicador permite a estimativa de efeitos diretos e indiretos de mortes ocorridas em epidemias/pandemias, auxilia o processo de tomada de decisão em diferentes fases do evento (9,10) e permite maior completude avaliativa das consequências negativas no sistema de saúde e na sociedade (10). 

No panorama nacional, fica evidente que cada região do país enfrentou realidades distintas em relação à pandemia, destacando-se a necessidade de abordagens diversas para compreender, de forma integrada e ampla, os impactos da covid-19 no Brasil, considerando a desigualdade regional que afeta o país.

Durante a pandemia, Pernambuco foi o estado brasileiro com o maior aumento na taxa de pobreza, registrando acréscimo de 8,14%, o que resultou em cerca de 1,6 milhão de pessoas passando a viver com a renda mensal inferior a R$ 500,00 (11). O estado foi o terceiro do Nordeste em número de casos confirmados (1.235.663) e óbitos pela covid-19 (23.241) (12), o que refletiu a complexidade dos impactos socioeconômicos e epidemiológicos da pandemia na região. No entanto, ainda são escassos estudos que avaliem o impacto da pandemia no território pernambucano, análise essencial para compreender os padrões da doença e suas consequências no estado e em outras regiões do Brasil. 

Este estudo objetivou estimar o excesso de óbitos durante a pandemia da covid-19 em Pernambuco entre 2020 e 2022. Para isso, comparou os óbitos ocorridos no estado no período 2020-2022 com a média dos últimos anos do período pré-pandêmico (2015-2019).

## Métodos

### Delineamento do estudo

Tratou-se de estudo descritivo que envolveu os óbitos notificados no Sistema de Informações sobre Mortalidade (SIM), entre 2015 e 2022, tendo como unidade de análise Pernambuco. Os dados encontravam-se atualizados até dezembro de 2022, consolidados no SIM até abril de 2024 e disponibilizados a partir de junho de 2024. Os dados foram extraídos em 15 de julho de 2024.

### Contexto

Pernambuco abrange 185 municípios, divididos em quatro macrorregiões de saúde: Região Metropolitana, Agreste, Sertão e Vale do São Francisco e Araripe. A secretaria estadual de saúde operacionaliza esses municípios em 12 regionais de saúde. Essas regionais estão sediadas em Recife (I Regional de Saúde), Limoeiro (II Regional de Saúde), Palmares (III Regional de Saúde), Caruaru (IV Regional de Saúde), Garanhuns (V Regional de Saúde), Arcoverde (VI Regional de Saúde), Salgueiro (VII Regional de Saúde), Petrolina (VIII Regional de Saúde), Ouricuri (IX Regional de Saúde), Afogados da Ingazeira (X Regional de Saúde), Serra Talhada (XI Regional de Saúde) e Goiana (XII Regional de Saúde) (13).

### Participantes

Foram incluídos todos os óbitos notificados no SIM entre 2015 e 2022, abrangendo todas as causas de morte, faixas etárias, de ambos os sexos, registradas em Pernambuco. 

Foram excluídos óbitos cujos registros apresentavam inconsistências ou ausência de informações essenciais, como dados de identificação das causas básicas ou associadas da morte. Óbitos ocorridos fora de Pernambuco ou não notificados no SIM não foram considerados na análise. 

### Variáveis analisadas

As variáveis analisadas incluíram dados relacionados aos óbitos gerais em Pernambuco e aos óbitos associados à covid-19, sendo estas: sexo (masculino, feminino), causa básica do óbito, ano e semana epidemiológica de ocorrência do óbito e regional de saúde de Pernambuco onde o óbito foi registrado.

A causa básica de morte foi analisada mediante a 10ª edição da Classificação Estatística Internacional de Doenças e Problemas Relacionados com a Saúde (CID-10), considerando todas as causas de morte descritas em seus 22 capítulos e abrangendo todas as categorias e todos os códigos registrados.

Os óbitos considerados como relacionados à covid-19 incluíram tantos aqueles em que a covid-19 foi registrada como a causa básica de morte quanto aqueles em que a covid-19 foi mencionada como causa contribuinte ou associada no atestado de óbito, sob a CID-10 B34.2 (infecção por coronavírus de localização não especificada) (14).

### Fontes de dados e mensuração

As informações sobre os óbitos foram obtidas no SIM. 

Para assegurar a robustez e transparência dos achados, foram utilizados os dados finais do SIM referentes ao período 2015-2022, consolidados até março de 2023. A coleta dos dados ocorreu somente após a disponibilização da versão final pelo SIM, garantindo a completude das informações e minimizando possíveis impactos de atrasos na notificação. Dessa forma, assegurou-se a exatidão dos resultados apresentados para o período analisado.

O excesso de óbitos foi medido como a diferença entre as mortes realmente relatadas durante determinado período em comparação com as mortes esperadas durante esse período se a pandemia de covid-19 não tivesse ocorrido. Empregou-se a seguinte fórmula: *Excesso de óbitos=número de mortes relatadas–número de mortes esperadas* (8,15).

Para efeito de comparabilidade, o P-score foi empregado em detrimento da apresentação do excesso de óbitos, visto que o número bruto de mortes em excesso dá a noção de escala, mas é menos comparável porque eles podem ter grandes diferenças no tamanho da população e nas mortes anuais esperadas.

Essa métrica é calculada como: *P-score=[(mortes relatadas–mortes projetadas)/mortes projetadas*]x100 (4).

A razão entre óbitos observados e óbitos esperados foi calculada para cada regional de saúde estudada (8). Esse cálculo propiciou análise detalhada das variações na mortalidade ao longo do tempo e em diferentes regiões do estado e contribuiu para a compreensão mais abrangente dos padrões de mortalidade durante o período em análise.

Para estimar a mortalidade esperada, foram usados os dados históricos de 2015 a 2019.

### Viés

Considerou-se a possibilidade de viés de informação, e isso aconteceu devido ao tipo de dado que este estudo empregou. O número relatado de mortes pode não contar todas as situações que ocorreram por limitações próprias da forma de se produzir a informação sobre a morte. Nem todos os locais têm capacidade para registrar e relatar adequadamente todas as mortes, devido à falta de hospitais, equipe de saúde e infraestrutura para registrá-las. No contexto da pandemia, os atrasos em relatórios de mortalidade foram frequentes, e os dados podem ser provisórios e incompletos nas semanas, nos meses ou mesmo nos anos após a ocorrência de uma morte.

Considerou-se também a possibilidade de viés causado pela incompletude de dados. Dados incompletos podem levar a conclusões erradas, redução da precisão e limitações na aplicabilidade dos resultados.

Outra limitação pode estar ligada a diferenças importantes entre as regionais de saúde, tais como infraestrutura da atenção à saúde, registros de óbitos e processos de vigilância. A pandemia acentuou as dificuldades operacionais tanto da atenção quanto do registro e investigação dos óbitos pela sobrecarga dos sistemas e profissionais responsáveis. Há de se considerar que a pandemia pode ter mascarado o efeito na mortalidade de outros eventos epidemiológicos (surtos, epidemias e endemias).

### Tamanho do estudo

O estudo foi censitário e não empregou cálculo amostral.

### Métodos estatísticos

A mortalidade esperada foi estimada a partir do modelo de regressão separadamente para cada regional de saúde e para todo o estado pernambucano: 

D_t_,_Y_= α_t_+β∙Y

Em que D_t_,_Y_ correspondeu ao número de mortes observadas durante uma semana t no ano Y; β correspondeu a uma inclinação linear ao longo dos anos; α_t_ foram interceptações separadas (efeitos fixos) para cada semana (mês/trimestre); e o ε ~ Ν(0,σ^2^) foi ruído gaussiano. Esse modelo conseguiu representar a variação sazonal na mortalidade e a tendência anual nos últimos anos devido à mudança na estrutura populacional ou nos fatores socioeconômicos (4).

Como o Brasil disponibiliza dados semanais, o modelo foi ajustado usando as semanas 1-52, uma vez que a semana 53 só acontecerá em alguns anos (incluindo 2020). Por isso, a linha de base para a semana 53 foi então tomada como igual ao valor obtido para a semana 52 (4). Para efeitos deste estudo, a mesma linha de base foi empregada para os anos estudados (2020-2022). 

A análise estatística dos dados foi realizada através do software R, versão 4.3.3. 

## Resultados

Demonstrou-se a frequência de óbitos em Pernambuco em nível de cada regional de saúde (Tabela 1). Durante o quinquênio que precedeu a eclosão da pandemia da covid-19 (2015-2019), observou-se a média de óbitos no estado correspondente a 63.778 (± 1.895). 

**Tabela 1:** Óbitos gerais e por covid-19 notificados por regional de saúde e ano. Pernambuco, 2015-2022

Regional de saúde	2015	2016	2017	2018	2019	2020		2021		2022	
						Geral	Covid-19	Geral	Covid-19	Geral	Covid-19
I – Recife	27.619	29.615	28.860	27.704	28.778	36.306	6.490	38.405	6.858	31.810	1.008
II – Limoeiro	4.242	4.354	4.375	4.219	4.329	4.882	578	5.314	838	4.746	120
III – Palmares	3.792	4.160	3.918	3.746	3.827	4.755	579	4.557	602	4.315	106
IV – Caruaru	9.991	10.601	9.743	9.399	9.618	10.899	1.314	11.202	1.563	10.991	407
V – Garanhuns	3.774	4.109	3.968	3.748	3.911	4.333	333	4.624	547	4.401	126
VI – Arcoverde	2.587	2.600	2.480	2.397	2.628	2.800	171	2.950	349	2.895	89
VII – Salgueiro	831	925	919	870	894	1.022	83	1.041	160	1.056	20
VIII – Petrolina	2.332	2.418	2.534	2.585	2.694	2.919	199	3.434	516	3.223	98
IX – Ouricuri	2.015	2.101	1.980	2.026	2.066	2.269	212	2.500	462	2.295	80
X – Afogados	1.348	1.475	1.369	1.356	1.430	1.605	144	1.708	227	1.579	43
XI – Serra Talhada	1.570	1.806	1.627	1.488	1.614	1.810	155	1.845	255	1.852	53
XII – Goiana	2.211	2.463	2.339	2.261	2.252	2.591	338	2.791	397	2.577	85
Total	62.312	66.627	64.112	61.799	64.041	76.191	10.596	80.371	12.774	71.740	2.235

A linha de base das mortes esperadas para Pernambuco em 2020, por meio da análise de regressão, foi 63.212 mortes. A partir desse cálculo, identificou-se que, nos anos estudados, o estado enfrentou contagem de morte de 20,9%, 27,5% e 13,8% maior que o previsto. O padrão evidenciado sobre os dados reflete que o ano de 2021 evidenciou proporcionalmente excesso de óbitos maior do que o ano de início da pandemia, em contraposição com a diminuição em 2022. Ao analisar esse comportamento por regional, evidenciou-se repetição dos padrões encontrados no estado, guardando as proporções devido ao tamanho populacional por regional (Figura 1).

**Figura 1:** Evolução dos óbitos gerais e devido à covid-19 por regionais de saúde. Pernambuco, 2015-2022

A razão entre óbitos observados e esperados foi maior nas regionais de saúde I e III em 2020; I e XII em 2021; e IV, VI, VII, VIII, XI e XII em 2022 (Tabela 2). Esses resultados indicaram mudança no padrão de distribuição do excesso de óbitos ao longo dos anos e evidenciaram o processo de interiorização das mortes. Essa informação foi reforçada pelos achados do P-score (Tabela 2). Os maiores excessos de mortalidade proporcional, disponibilizados pelo P-score, foram observados nas regionais de saúde I, III e IV em 2020; I, XII e VIII em 2021; e XI, IV e VII em 2022.

**Tabela 2:** Razão entre óbitos anuais observados e esperados e P-score. Pernambuco, 2020-2022

Regional de saúde	Razão de mortes observadas e esperadas			P-score		
	2020	2021	2022	2020	2021	2022
I – Recife	1,3	1,4	1,1	28,0	35,4	12,2
II – Limoeiro	1,1	1,2	1,1	13,2	23,2	10,0
III – Palmares	1,3	1,2	1,1	25,9	20,7	14,3
IV – Caruaru	1,2	1,2	1,2	18,1	21,4	19,1
V – Garanhuns	1,1	1,2	1,1	11,4	18,9	13,2
VI – Arcoverde	1,1	1,2	1,2	11,0	17,0	14,8
VII – Salgueiro	1,1	1,2	1,2	12,5	14,6	16,3
VIII – Petrolina	1,1	1,2	1,2	4,8	23,3	15,7
IX – Ouricuri	1,1	1,2	1,1	11,4	22,7	12,7
X – Afogados	1,2	1,2	1,1	15,6	23,0	13,7
XI – Serra Talhada	1,2	1,2	1,2	17,3	19,6	20,1
XII – Goiana	1,2	1,3	1,2	15,7	24,7	15,1
Total	1,2	1,3	1,1	20,9	27,5	13,8

Verificou-se a distribuição de óbitos entre a população masculina e a população feminina considerando as causas e as regionais de saúde, em que a maior frequência sempre se apresentou sobre a população masculina (Tabela 3).

**Tabela 3:** Distribuição por sexo das mortes gerais e por covid-19 relatadas por regional de saúde e ano. Pernambuco, 2020-2022

Regional de saúde	2020		2021		2022	
	Geral	Covid-19	Geral	Covid-19	Geral	Covid-19
**População masculina**						
I – Recife	19.303	3.451	19.973	3.591	16.618	513
II – Limoeiro	2.784	317	2.891	436	2.578	61
III – Palmares	2.737	330	2.529	319	2.403	51
IV – Caruaru	6.007	717	6.156	822	6.010	216
V – Garanhuns	2.412	175	2.583	300	2.367	63
VI – Arcoverde	1.568	86	1.661	191	1.601	55
VII – Salgueiro	602	38	584	93	567	11
VIII – Petrolina	1.686	119	1.994	287	1.854	56
IX – Ouricuri	1.273	130	1.414	247	1.338	49
X – Afogados	893	82	936	123	898	25
XI – Serra Talhada	997	79	1.033	136	1.036	25
XII – Goiana	1.494	189	1.556	200	1.465	40
Total	41.756	5.713	43.310	6.745	38.735	1.165
**População feminina**						
I – Recife	16.991	3.039	18.425	3.267	15.185	495
II – Limoeiro	2.098	261	2.423	402	2.167	59
III – Palmares	2.016	249	2.027	283	1.912	55
IV – Caruaru	4.889	597	5.039	741	4.975	191
V – Garanhuns	1.920	158	2.039	247	2.034	63
VI – Arcoverde	1.229	85	1.288	158	1.293	34
VII – Salgueiro	420	45	457	67	489	9
VIII – Petrolina	1.232	80	1.436	229	1.368	42
IX – Ouricuri	996	82	1.084	215	956	31
X – Afogados	712	62	772	104	681	18
XI – Serra Talhada	812	76	811	119	816	28
XII – Goiana	1.097	149	1.234	197	1.112	45
Total	34.412	4.883	37.035	6.029	32.988	1.070

A razão de óbitos observados e esperados no sexo masculino, em relação às regionais de saúde, apresentou padrão similar à de Pernambuco, excetuando-se em 2021, em que apresentou maiores razões para as regionais I, V e IX (Tabela 4). O maior excesso de óbitos, conforme indicado pelo P-score, foi registrado para as regionais I, III e VII em 2020; I, IX e V em 2021; e XI, IX e VI em 2022. No último ano, todas as regionais com os valores mais altos estavam localizadas no Sertão do estado. Para a população masculina, a VIII Regional de Saúde (Petrolina) apresentou menor proporção de excesso de óbitos. 

**Tabela 4:** Distribuição por sexo da razão entre mortes observadas e esperadas por ano e P-score. Pernambuco, 2020-2022

Regional de saúde	Razão de mortes observadas e esperadas			P-score		
	2020	2021	2022	2020	2021	2022
**População masculina**						
I – Recife	1,3	1,3	1,1	28,5	33,0	10,7
II – Limoeiro	1,1	1,2	1,1	14,4	18,8	6,0
III – Palmares	1,3	1,2	1,1	25,3	15,7	10,0
IV – Caruaru	1,2	1,2	1,2	17,0	19,9	17,1
V – Garanhuns	1,2	1,3	1,1	16,3	24,5	14,1
VI – Arcoverde	1,2	1,2	1,2	14,9	21,7	17,3
VII – Salgueiro	1,2	1,2	1,1	20,7	17,1	13,7
VIII – Petrolina	1,0	1,2	1,1	3,9	22,9	14,3
IX – Ouricuri	1,1	1,3	1,2	12,3	24,7	18,0
X – Afogados	1,1	1,2	1,1	11,8	17,2	12,4
XI – Serra Talhada	1,2	1,2	1,2	16,8	21,1	21,4
XII – Goiana	1,2	1,2	1,2	17,1	22,0	14,9
Total	1,2	1,2	1,1	20,7	25,2	12,0
**População feminina**						
I – Recife	1,3	1,4	1,1	27,5	38,2	13,9
II – Limoeiro	1,1	1,3	1,2	11,8	29,1	15,4
III – Palmares	1,3	1,3	1,2	26,8	27,5	20,3
IV – Caruaru	1,2	1,2	1,2	19,6	23,3	21,7
V – Garanhuns	1,1	1,1	1,1	5,8	12,4	12,1
VI – Arcoverde	1,1	1,1	1,1	6,4	11,5	11,9
VII – Salgueiro	1,0	1,1	1,2	2,6	11,6	19,4
VIII – Petrolina	1,1	1,2	1,2	5,9	23,5	17,6
IX – Ouricuri	1,1	1,2	1,1	10,4	20,1	5,9
X – Afogados	1,2	1,3	1,2	20,7	30,9	15,4
XI – Serra Talhada	1,2	1,2	1,2	17,9	17,8	18,5
XII – Goiana	1,1	1,3	1,2	13,9	28,1	15,5
Total	1,2	1,2	1,1	20,3	29,5	15,4

Para o sexo feminino, a razão de óbitos apresentou comportamento muito similar ao observado para a população geral, com exceção de 2022. Em relação ao P-score, as maiores proporções de excesso de óbitos foram identificadas nas regionais de saúde I, III e X em 2020; I, X e II em 2021; e IV, III e VII em 2022 (Tabela 4).

## Discussão

A pandemia da covid-19 trouxe grandes impactos para a saúde pública, sendo notório que o período correspondente ao pico de incidência dos casos gerou aumento do número de óbitos no panorama global (2). Nesse contexto, é atribuído ao Brasil o segundo lugar em relação ao número de óbitos decorrente da doença, sendo precedido pelos Estados Unidos (3).

Este estudo, realizado em Pernambuco, detectou excesso de óbitos no período analisado, fato atribuído, sobretudo, à pandemia de covid-19 que surgiu como novo fator para mortalidade. Esses dados corroboram estudos ecológicos realizados em outras regiões do país (8,14).

Identificou-se neste estudo que os dois primeiros anos da pandemia apresentaram aumento nos óbitos significativo para as regionais de Pernambuco, geralmente com padrão de ascensão. Houve exceção com relação à regional III (Palmares), que, por todo o período da pandemia, apresentou diminuição no número de óbitos após 2020. Esse resultado está alinhado às fases mais críticas da pandemia, caracterizadas por altas taxas de transmissão, surgimento de novas variantes e sobrecarga nos sistemas de saúde (16).

O excesso de mortalidade é uma medida mais abrangente do impacto total da pandemia na mortalidade, em comparação com o número de mortes confirmadas por covid-19. Isso ocorre porque ele captura não apenas mortes confirmadas, mas também as que não foram diagnosticadas e relatadas com precisão, bem como mortes por outras causas que são atribuíveis a interrupções e condições gerais de crise (8,14,15). 

Após o período crítico da pandemia, evidenciou-se que, em 2022, o excesso de óbitos decaiu de forma significativa, excesso pelas regionais VII e XI (Salgueiro e Serra Talhada). Essa diminuição possivelmente foi resultante da vacinação escalonada, com a priorização da imunização de pessoas pertencentes aos grupos com maior risco de morbimortalidade (17), cabendo-se ainda buscar o entendimento para as regionais citadas terem mantido o padrão de crescimento. 

Observou-se que a razão entre óbitos observados e esperados foi maior nas regionais de saúde I e III em 2020, I e XII em 2021 e IV, VI, VII, VIII, XI e XII em 2022 (Tabela 2). Esses resultados indicaram mudança no padrão de distribuição do excesso de óbitos ao longo dos anos e evidenciaram o processo de interiorização das mortes, demonstrando o deslocamento progressivo do impacto da pandemia das regiões metropolitanas para áreas interioranas, relacionado ao aumento da mobilidade populacional, à menor infraestrutura de saúde e às dificuldades na implementação de medidas de controle e vigilância (8).

Ao analisar a linha do tempo do excesso de óbitos provocado pela covid-19, em Pernambuco, notou-se maior ocorrência de mortes na Região Metropolitana em 2020, ratificando estudos que mostraram maior incidência de infecção em regiões mais densamente povoadas (18). Contudo, em 2021, houve intensificação das mortes no interior do estado, principalmente na área correspondente às VIII e IX regionais de saúde. Essa interiorização reflete uma característica própria do interior de Pernambuco, com áreas que abrigam populações em situação de vulnerabilidade e extrema pobreza, mas que apresentam grandes impasses para serem alcançadas por políticas públicas (18). 

Tratando-se especificamente das VIII e IX regionais de saúde, um fator condicionante possível para a elevação do excesso de óbitos foi o crescimento populacional ocorrido nos últimos anos, visto que, no Censo Demográfico de 2010, a população adscrita na VIII Regional de Saúde era de 434.713,0 habitantes e, no Censo Demográfico de 2022, passou a ser de 531.221,0, o que representou o acréscimo de 96.508,0 (22,2%). Houve aumento populacional também na IX Regional de Saúde, de 328.173 para 338.050 habitantes, o que representou o aumento de 9.877 (3,0%). 

A comparação do número de óbitos por covid-19 entre as diferentes regiões tem sido desafiador, porque esses podem ser afetados pela capacidade de teste de cada local e pela política de notificação (19). Contudo, estudos ecológicos globais demonstraram como fatores ambientais, demográficos e geográficos, além da dimensão político-legal, contribuem na ocorrência e morbimortalidade da covid-19 (20). 

Ainda que as áreas territoriais do Sertão e do São Francisco e Vale do Araripe sejam maiores que a da Região Metropolitana, faz-se necessário considerar que a densidade demográfica e o processo de urbanização desacelerado interferiram na realidade epidemiológica do interior do estado, mesmo com a menor quantidade e qualificação dos equipamentos de saúde. Entretanto, a interpretação desses resultados requer cautela, pois projeções baseadas em tendências lineares podem superestimar ou subestimar os valores reais, especialmente em regiões com crescimento populacional acelerado (21). 

Os achados deste estudo identificaram que o excesso de óbitos foi maior entre homens, se comparado ao que foi observado entre mulheres, assim como observado anteriormente (1,22).

A mortalidade por covid-19 tende a ser maior no sexo masculino, o que corrobora os resultados deste estudo, e pode ser explicada pela combinação de fatores imunológicos, genéticos, hormonais e sociais (22). Do ponto de vista imunológico, os homens possuem menores níveis de imunoglobulina G na fase inicial da doença, o que pode contribuir para piores desfechos (23). Geneticamente, o cromossomo X expressa genes essenciais para a imunidade, incluindo aqueles envolvidos na sinalização de citocinas e receptores Toll-like, além do gene ACE2, receptor do coronavírus tipo 2, cuja expressão pode ser influenciada pelo escape da inativação do X, resultando em diferenças sexuais na expressão do ACE2 e aumentando a vulnerabilidade ao vírus (24,25). Quanto aos hormônios sexuais masculinos, embora os efeitos ainda sejam incertos, baixos níveis de testosterona têm sido associados a piores desfechos clínicos, pelo papel desse hormônio na regulação da inflamação e influência na expressão de TMPRSS2, uma protease essencial para a entrada viral (26,27). Socialmente, as influências de gênero podem atuar como modificadores sociais e psicológicos da apresentação da doença, com destaque para a menor busca por serviços de saúde e a baixa adesão ao autocuidado entre os homens (28).

Este estudo enfrentou limitações inerentes ao uso de dados secundários, como subnotificação de óbitos e possíveis vieses relacionados à variabilidade de capacidade diagnóstica e de notificação, bem como decorrentes de diferenças infraestruturais entre regiões. Isso pode distorcer as comparações do excesso de óbitos entre as regionais de saúde, de forma semelhante ao observado em outros estudos nacionais (28,29).

Outras possíveis limitações decorreram da falta de controle de fatores sazonais ou epidemias sobrepostas que possam ter contribuído para o aumento do número de óbitos em determinados períodos. A ausência de estratificação por faixas etárias e grupos de risco também pode ocultar diferenças importantes no padrão de mortalidade, sendo necessários estudos futuros para preencher essas lacunas.

O monitoramento contínuo de casos e a vigilância em saúde, especialmente no âmbito epidemiológico, são essenciais para a proteção da população, pois permitem detectar precocemente surtos, identificar fatores de risco, analisar tendências e implementar intervenções rápidas e eficazes que previnem a propagação de enfermidades e reduzem fatalidades (5), sendo fundamentais no contexto da covid-19. 

Diante dos dados apresentados, afirma-se que a covid-19 teve impacto relevante na saúde da população de Pernambuco, com excesso de óbitos significativo nos períodos mais críticos da pandemia, momento no qual, devido ao colapso dos sistemas de saúde, ocorreram óbitos direta e indiretamente atribuíveis à covid-19. O excesso de óbitos é indicador essencial para monitorar os impactos de novas emergências de saúde, visando definir prioridades e medidas de saúde pública adequadas, sobretudo em regiões onde ainda há deficiências em sistemas para diagnóstico e notificações eficientes.

Futuros estudos podem explorar análises mais aprofundadas ao incorporar dados geoespaciais, padrões de mobilidade populacional e efeitos a longo prazo da pandemia, tanto na mortalidade quanto nas condições crônicas de saúde.

## Data Availability

Os bancos de dados utilizados na pesquisa estão disponíveis em: 0.5281/zenodo.13738412.
